# Special Issue “Diet and Lifestyle: Impact on the Molecular and Cellular Mechanism of NCDs”

**DOI:** 10.3390/ijms26199589

**Published:** 2025-10-01

**Authors:** Elena Azzini, Valeria Gasperi, Angela Polito

**Affiliations:** 1 CREA-Research Centre for Food and Nutrition, Via Ardeatina 546, 00178 Rome, Italy; angela.polito@crea.gov.it; 2 Department of Experimental Medicine, University of Rome “Tor Vergata”, 00133 Rome, Italy

The global health risks associated with the ongoing crisis caused by the SARS-CoV-2 virus have profoundly shifted our thinking. It has reminded the world that acute infections do not occur in isolation but rather intersect with the ongoing challenges posed by non-communicable diseases (NCDs) [1]. Rather than a simple ‘twofold burden’, what became evident was a dynamic interaction in which infections, metabolic disorders, and chronic low-grade inflammation reinforce one another. This interplay was particularly evident in individuals with obesity, diabetes, cardiovascular disease and other NCDs, who were at a significantly higher risk of severe outcomes from the virus [2]. This evidence clearly shows that prevention strategies must encompass not only infectious disease control, but also diet, lifestyle, and their biological impact at molecular and cellular levels [3].

This Special Issue brings together a series of original studies and reviews exploring how nutrition and lifestyle influence the mechanisms linking metabolic balance, inflammation and tissue resilience. The articles demonstrate that the effects of diet extend far beyond the supply of calories, influencing immunity, the composition of the gut microbiota, musculoskeletal health, and organ function.

Chlorella vulgaris is a green alga that has gained popularity as a dietary supplement due to its nutritional content [4,5,6]. It is thought to offer antioxidant, immune-boosting, lipid-lowering and detoxifying effects, making it a promising candidate for nutritional research. Studies have explored its impact on health, immune function, and nutrition [7,8]. One study found that taking Chlorella vulgaris supplements can have a beneficial effect on the gut microbiome by enhancing the production of short-chain fatty acids and limiting harmful bacterial populations. This supports intestinal and systemic health (contribution 1).

Alterations to the microbiome and liver damage can be caused by weight cycling and appear to be only partially reversible with weight loss [9]. Weight cycling, involving repeated weight loss and regain, affects most individuals attempting to lose weight, with only around 20% achieving long-term maintenance [10,11]. The impact of yo-yo dieting remains debated, though some studies suggest that weight regain may worsen liver fat accumulation, inflammation and microbiome alterations compared to stable obesity [12,13]. Another study using a zebrafish model of weight cycling revealed tissue-specific trade-offs associated with fluctuating calorie intake. While repeated dieting temporarily reduced fatty liver, it ultimately worsened hepatic pathology, even though muscle function remained intact (contribution 2).

As is well known, a well-structured diet supports health and longevity and reduces the risk of age-related diseases. The quality and source of protein, including its amino acid composition, are particularly important for muscle health and overall well-being in older adults [14,15]. Both plant- and animal-based proteins offer benefits, though their respective impacts differ and the optimal balance remains to be investigated. A human study on protein intake further emphasized the importance of both quantity and source: higher consumption of plant protein (≥40%) was associated with a lower risk of non-communicable diseases (NCDs) in older adults, with age- and sex-dependent effects on inflammatory cytokine regulation (contribution 3).

There is a close interconnection between the gut microbiome, bone health, interleukins, chronic periodontitis, and the impact of the SARS-CoV-2 virus on the body. This highlights the importance of considering genetic predispositions and immune dynamics when predicting disease outcomes [16]. The intricate link between gut function and bone development was demonstrated in a zebrafish model, in which soy-induced intestinal inflammation impaired bone formation and mineralization. This demonstrates how gastrointestinal health can influence skeletal integrity (contribution 4).

In recent decades, the global increase in obesity, which is linked to lifestyle changes, has led to an increase in metabolic syndrome. Through lipotoxicity and altered lipid metabolism, metabolic syndrome contributes to the development of chronic kidney disease [17,18,19]. However, the early effects of these alterations on the kidneys remain unclear, and treatment options for diabetic nephropathy are still limited. In this context, curcumin emerges as a promising alternative strategy [20]. Furthermore, research on bisdemethoxycurcumin (BDMC), a turmeric-derived compound, revealed its ability to counteract chronic kidney disease caused by high-fat diets by activating the Keap1/Nrf2 pathway and reducing oxidative stress-related damage (contribution 5).

Taken together, these contributions highlight that food is much more than just a source of calories and nutrients. Dietary components act as bioactive regulators of inflammation, oxidative balance, microbial ecology and tissue homeostasis. They also highlight the importance of factors such as sex, age, and long-term adherence in determining outcomes, challenging overly simplistic models of diet–disease relationships.

## Conclusions

In conclusion, the contributions to this Special Issue reinforce the idea that preventing and managing non-communicable diseases (NCDs) requires an understanding of how diet and lifestyle affect molecular and cellular health. This body of work provides new insights into how we might mitigate the growing burden of NCDs and promote healthier, more sustainable lifestyles in the post-pandemic era by integrating nutritional biochemistry, experimental models, and human studies.

We thank all the authors and reviewers for their valuable contributions and look forward to further advancing this critical field.
